# CalDAG-GEFI Deficiency in a Family with Symptomatic Heterozygous and Homozygous Carriers of a Likely Pathogenic Variant in *RASGRP2*

**DOI:** 10.3390/ijms222212423

**Published:** 2021-11-17

**Authors:** Sara Morais, Mónica Pereira, Catarina Lau, Ana Gonçalves, Catarina Monteiro, Marta Gonçalves, Jorge Oliveira, Lurdes Moreira, Eugénia Cruz, Rosário Santos, Margarida Lima

**Affiliations:** 1 Setor de Trombose e Hemostase, Serviço de Hematologia Clínica, Hospital de Santo António (HSA), Centro Hospitalar Universitário do Porto (CHUPorto), 4099-001 Porto, Portugal; mpereira.hematologiaclinica@chporto.min-saude.pt (M.P.); catarina.monteiro2409@gmail.com (C.M.); lurdesamoreira@gmail.com (L.M.); eugeniacruz.hematologiaclinica@chporto.min-saude.pt (E.C.); 2 UMIB—Unidade Multidisciplinar de Investigação Biomédica, ICBAS—Instituto de Ciências Biomédicas Abel Salazar, Universidade do Porto, 4050-131 Porto, Portugal; catarinalau.hematologiaclinica@chporto.min-saude.pt (C.L.); ana.goncalves@chporto.min-saude.pt (A.G.); martagoncalves.hematologiaclinica@chporto.min-saude.pt (M.G.); rosario.santos@chporto.min-saude.pt (R.S.); margaridalima@chporto.min-saude.pt (M.L.); 3 Laboratório de Citometria, Serviço de Hematologia Clínica, Hospital de Santo António (HSA), Centro Hospitalar Universitário do Porto (CHUPorto), 4099-001 Porto, Portugal; 4 Unidade de Genética Molecular, Centro de Genética Médica Doutor Jacinto Magalhães (CGMJM), Centro Hospitalar Universitário do Porto (CHUPorto), 4050-466 Porto, Portugal; jorgemsmoliveira@gmail.com

**Keywords:** platelets, *RASGRP2*, CalDAG-GEFI deficiency, platelet function diseases

## Abstract

*RASGRP2* encodes the calcium and diacylglycerol (DAG)-regulated guanine nucleotide exchange factor I (CalDAG-GEFI) identified as a Rap1-activating molecule. Pathogenic variants previously identified in *RASGRP2* allowed the characterization of CalDAG-GEFI deficiency as a non-syndromic, autosomal recessive platelet function disease. We report on the clinical manifestations and laboratory features of a Portuguese family with a likely pathogenic variant in *RASGRP2* (c.999G>C leading to a p.Lys333Asn change in the CDC25 catalytic domain of CalDAG-GEFI) and discuss the contribution of this variant to the disease manifestations. Based on the study of this family with one homozygous patient and five heterozygous carriers and on a critical analysis of the literature, we challenge previous knowledge that CalDAG-GEFI deficiency only manifests in homozygous patients. Our data suggest that at least for the *RASGRP2* variant reported herein, there is a phenotypic expression, albeit milder, in heterozygous carriers.

## 1. Introduction

In 2014, Canault et al. described, for the first time, the association between severe bleeding caused by platelet (PLT) dysfunction and pathogenic variants in the *RASGRP2* gene in humans [1]. Since then, several other *RASGRP2* variants have been reported worldwide [2]. *RASGRP2* encodes the calcium and diacylglycerol (DAG)-regulated guanine nucleotide exchange factor I (CalDAG-GEFI) identified as a Rap1-activating molecule. Rap1 is one of the main small GTPases in platelets and constitutes a key signaling element that directs platelet activation by regulating integrin-mediated aggregation and granule secretion [3]. DAG and calcium activate many isoforms of platelet protein kinase C (PKC) and CalDAG-GEFI, leading to the conversion of Rap1-GDP to Rap1-GTP and its translocation to the plasma membrane [4]. During platelet activation, Talin is recruited from the cytoplasm to αIIbβ3 in a process that is regulated, in part, by the Rap1 GTPase [3]. The role of CalDAG-GEFI in calcium-dependent PLT activation was established in a *CalDAG-GEFI^-/-^* mouse model, in which induction of inside-out activation of integrin αIIbβ3 was strongly inhibited [5].

Patients with biallelic *RASGRP2* variants have been described to have moderate to severe bleeding, normal PLT count and morphology, reduced PLT aggregation in response to ADP and epinephrine, and variable aggregation defects with other agonists [6]. In addition, they show impaired αIIbβ3 integrin activation, as evaluated by the exposure of PAC-1 epitopes and fibrinogen binding in response to most agonists except phorbol 12-myristate 13-acetate (PMA) [6]. The review of pathogenic variants in *RASGRP2* classifies CalDAG-GEFI deficiency as a non-syndromic, autosomal recessive (AR) platelet function disease [6,7].

Herein we report a family with a likely pathogenic variant in *RASGRP2,* in which the clinical manifestations and laboratory results have documented phenotypic expression, albeit milder, in heterozygous carriers.

## 2. Results

**Figure 1:**
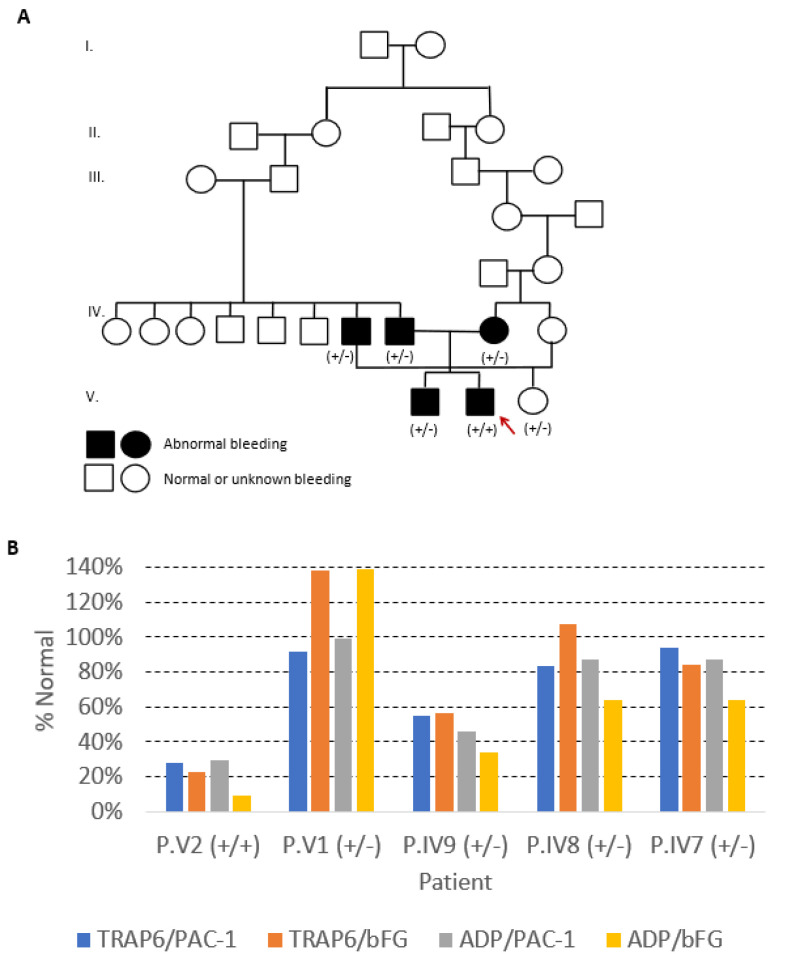
(**A**) Family pedigree of the index case (V2) with a homozygous likely pathogenic variant in *RASGRP2*. The black symbols indicate cases with abnormal bleeding and the white symbols indicate family members without or unknown bleeding symptoms. The index case is signaled with an arrow. +/−, heterozygous; +/+, homozygous for the variant. (**B**) Platelet αIIbβ3 integrin activation after stimulation with the agonists thrombin receptor-activating peptide-6 (TRAP6) and adenosine diphosphate (ADP), as evaluated by flow cytometry using fluorescein-conjugated PAC-1 and anti-bound fibrinogen (bFG) monoclonal antibodies (mAbs). The results are presented as percentages of the normal values, by dividing the median value of the fluorescence intensity of the patientߣs platelets stained with PAC-1 and anti-bFG mAbs after stimulation with TRAP6 or with ADP by the median of correspondent values obtained in controls.

The index case, Patient V2, bleeding score (BS) of 8, a 12-year-old boy from a consanguineous Portuguese family (Figure 1A), was studied in 2011 at 4 years old, because of mucocutaneous bleeding. He had been hospitalized at the age of two, due to severe epistaxis requiring nasal packing and red blood cell transfusion. He also presented easy bruising and gingival bleed. His brother (P.V1, BS: 5), a 16-year-old boy, was studied at 7 years of age, because of bleeding during tonsillectomy; he also bled after deciduous tooth extraction. His mother (P.IV9, BS: 6), a 37-year-old woman, reported epistaxis during childhood, easy bruising, and gingival bleeding; however, she had two cesarean deliveries and an ophthalmic surgery without hemorrhage. His father (P.IV8, BS: 6), deceased at age 48 with non-traumatic intracranial hemorrhage, had frequent epistaxis not requiring medical intervention. His paternal uncle (P.IV7, BS: 7), a 53-year-old man, also had clinically relevant bleeding: epistaxis with the need of cauterization, bleeding from minor wounds, gingival bleed, and hemorrhage after dental extraction. On the other hand, his cousin (Patient V3, BS: 2), a 20-year-old female, daughter of P. IV7, had no bleeding symptoms, although she never had surgery. None of the patients showed increased susceptibility to infections. Other family members were not available for clinical or laboratorial evaluation.

Laboratory studies of the index case showed normal PLT counts and blood coagulation tests, extended closure time in PFA-200, and normal PLT glycoprotein expression as evaluated by flow cytometry (FCM) (Table 1). PLT aggregations were almost or even absent in response to adenosine diphosphate (ADP) (10 µM), epinephrine (EPI) (10 µM), and arachidonic acid (AA) (1 mM), decreased in response to collagen (COL) (1 µg/mL), but normal in response to thrombin receptor-activating peptide–6 (TRAP-6) (25 µM); ristocetin (RIST) (1 mg/mL) induced PLT agglutination was reduced. Adenosine triphosphate (ATP) release was also impaired with ADP, EPI, and AA (Table 1). In addition, αIIbβ3 integrin activation, as evaluated by FCM using fluorescein-conjugated PAC-1 and anti-bound fibrinogen (bFG) monoclonal antibodies (mAbs), was significantly reduced (<30% of the normal) after stimulation with TRAP-6 and ADP (Table 1 and Figure 1B).

**Abbreviations:** %N, % of the normal values; % (nmol), % of aggregation and nmol of released ATP; AA, arachidonic acid; ADP, adenosine diphosphate; aPTT, activated partial thromboplastin time; BS, bleeding score; bFG, bound fibrinogen; COL, collagen; COL/ADP, collagen/adenosine diphosphate; COL/EPI, collagen/epinephrine; CT, closure time; EPI, epinephrine; FCM, flow cytometry; FSC, forward scatter; FVIII:C, Factor VIII procoagulant activity; GP, glycoprotein; Hb, hemoglobin; ISTH-BAT, International Society on Thrombosis and Hemostasis-Bleeding Assessment Tool; MPV, mean platelet volume; NA, not available; PFA, platelet function assay; PLT, platelet; PT, prothrombin time; RIST, ristocetin; SSC, side scatter; TRAP-6, thrombin receptor-activating peptide-6; WBC, white blood cells; vWF:Ag, von Willebrand factor antigen; vWF:RCo, ristocetin cofactor activity of von Willebrand factor.

**Normal ranges:** BS (ISTH-BAT): <4 in adult males, <6 in adult females and <3 in children; ATP release: COL: 0.6–2.3 nmol; ADP: 0.3–2.6 nmol; AA: 0.6–2.3 nmol; EPI: 0.5–2.5 nmol; TRAP6: 0.7–3.4 nmol; PLT aggregation: COL: 60–112%; ADP: 61–100%; AA: 62–105%; EPI: 56–112%; TRAP6: 70–113%; PLT counts: 150–400 × 10^9^/L; MPV: 7.2–11.1 fL; PLT closure times: COL/ADP: 88–146 s; COL/EPI: 56–120 s; PLT glycoproteins: CD41a: 85–115%; CD61: 80–120%; CD42b: 70–130%; PLT mepacrine uptake: 89–122%

Laboratory studies of the symptomatic heterozygous relatives also showed normal PLT counts and blood coagulation parameters but variable PFA-200 closure times and PLT aggregation tests (Table 1). This variability was among patients and, for the same individual, between different experiments. The closure time was prolonged in patient IV9 and normal for the other relatives studied. All relatives but one (P.V1) showed severely decreased PLT aggregation in response to EPI and normal PLT aggregation in response to COL, AA, and TRAP-6. The mother (P.IV9) showed the most impaired PLT aggregation pattern, having also a variable response to ADP (absent or diminished in two different experiments) and diminished ristocetin-induced agglutination. Patient V1 showed normal aggregation tests. ATP release in response to ADP and EPI was also diminished, though with variable response according to the agonist and the patient studied (Table 1). Platelet αIIbβ3 integrin activation yielded variable results, being reduced to about half of the normal in P.IV9 (mother) after stimulation with TRAP-6 and ADP, and to about 60% after stimulation with ADP in P.IV7 (paternal uncle) and P.IV8 (father); P.V1 (brother) showed normal αIIbβ3 integrin activation with both agonists (Table 1 and Figure 1B). Laboratory studies of the asymptomatic heterozygous relative (V3) were normal (Table 1).

Deoxyribonucleic acid (DNA) analysis of the index case using the ThromboGenomics high-throughput sequencing (HTS) platform identified a novel homozygous variant in exon 9 of the *RASGRP2* gene (NM_153819.1:c.999G>C), leading to an amino acid change (p.Lys333Asn) in the CDC25 catalytic domain of CalDAG-GEFI. We confirmed through Sanger sequencing that the index patient was homozygous for the c.999G>C variant and that all the five studied relatives were heterozygous for the variant. This variant has not been previously reported in the literature or in the gnomAD/ExAC databases. Bioinformatic analyses suggest a deleterious effect. DNA analysis of the proband’s mother (P.IV9) performed in our hospital using a different HTS panel, covering 92 genes known to be associated with hemostasis disorders, confirmed heterozygosity for the c.999G>C variant found in the proband (P.V2), and revealed a heterozygous intronic variant in the *RGS2* gene (c.212+3_212+6del); this intronic variant was present in the proband (P.V2) and in his mother (P.IV9) but not in the other family members (P.IV7, P.IV8, P.V1), and bioinformatic analyses suggest a variant of uncertain significance (VUS).

## 3. Discussion

Our index case shares clinical characteristics with the other cases previously described [7]: a predominantly mucocutaneous bleeding disorder diagnosed during childhood.

In most of the previously reported families, the monoallelic carriers were considered asymptomatic or not described. In our family, all the carriers but one had mild-to-moderate bleeding: almost all had epistaxis of variable severity, and two patients had abnormal bleeding following surgery or dental extraction. Curiously, the abnormal bleeding due to surgery occurred in patient V1, without impaired functional studies. We can speculate that P.V1 with normal platelet aggregation may have compromised adhesion and spreading, similarly to other heterozygous patients previously described [1]. It should be noted that this patient only bled in surgery, and the only asymptomatic relative (V3) has never undergone surgery. Heterozygous carriers appear to experience bleeding whenever they undergo hemostatic challenges such as surgeries, as in other mild-to-moderate PLT function defects.

Surprisingly, from the literature available, only Canault et al. have described the PLT function in heterozygous carriers [1], reporting pronounced defects in adhesion under flow and spreading. According to Canault, the presence of only one normal allele is sufficient to prevent bleeding and to support normal PLT aggregation but not full adhesion and spreading [1]. In our family, symptomatic heterozygous carriers have bleeding symptoms, but their PLT function is impaired still to a lesser extent than in the homozygous patient.

Before the first description of CalDAG-GEFI deficiency in humans, Puetz and Boudreaux studied its encoding gene in patients with mucocutaneous bleeding and impaired PLT reactivity that could not be explained by known causes of acquired or inherited PLT dysfunction [8]. Three patients, all with an impaired aggregation response, were found to have single nucleotide polymorphisms (SNP) in the coding region of *RASGRP2*, predicted to result in amino acid changes; however, all these patients were heterozygous for the SNP identified. One of these SNP was also found in normal control. These data agree with the findings in our family in which heterozygous patients present variable bleeding symptoms and variable impaired PLT aggregations, but they can also be asymptomatic.

CalDAG-GEFI deficiency has been assumed as an AR disease by the analysis of several cases reported in the literature, with heterozygous carriers consistently considered asymptomatic and displaying no major PLT dysfunction [6]. Upon reviewing these reports, however, where all the patients studied were homozygous for variants in *RASGRP2,* it appears that their relatives were not evaluated by functional studies, except in the family reported by Canault [1], which is perhaps because the monoallelic carriers may have a mild or even absent bleeding, which went disregarded when compared with the more severe hemorrhage patterns displayed by the biallelic carriers.

In contrast, all but one heterozygous carriers of our family had a clear bleeding phenotype and displayed PLT dysfunction. We can speculate if it is due to a different variant in *RASGRP2* with a more severe phenotype, or to the presence of a co-existing disorder that made them more likely to have clinical signs. Although we cannot completely exclude the presence of a second modifier variant, the DNA analysis of the proband and his mother by HTS minimizes this possibility. The variant identified in the *RGS2* gene, classified as VUS, present only in the proband (P.V2) and the mother (P.IV9), does not seem to explain the hemorrhagic phenotype, nor the defective platelet function in the family.

The unexpected result of impaired platelet agglutination with ristocetin was accompanied by the normal expression of GPIbIX complex components and plasmatic levels of von Willebrand Factor, which exclude the coexistence of von Willebrand disease or Bernard–Soulier syndrome. We can speculate if abnormalities in GPIbIX function are secondary to integrin defects, as previously hypothesized in other analogous disorders of αIIbβ3 integrin signaling [9]. Curiously, these results are as other results recently described in a family with a homozygous variant in *RASGRP2* considered as causative of the bleeding phenotype [10].

The presence of symptomatic monoallelic carriers in our family, together with the previous findings of heterozygous exonic variants (SNPs) described in symptomatic patients with impaired PLT reactivity [8] and defects in PLT adhesion and spreading that were previously described in heterozygous carriers of another *RASGRP2* pathogenic variant [1], suggested a likely autosomal dominant (AD) mode of inheritance for the CalDAG-GEFI deficiency. Based on these findings, it is realistic to assume that CalDAG-GEFI deficiency is a genetic heterogeneous trait with variable expressivity.

Nowadays, the modes of inheritance for different congenital PLT diseases are being debated. Recent reports of AR platelet disorders found milder phenotypes in the obligate carriers [11]. It is possible that CalDAG-GEFI deficiency, beyond the previously proposed AR mode of inheritance, may present an AD mode of inheritance, with a milder phenotype in heterozygous carriers of *RASGRP2* variants.

## 4. Material and Methods

### 4.1. Clinical Evaluation

Patients underwent a clinical appointment where the BS was determined using the International Society on Thrombosis and Hemostasis (ISTH)—Bleeding Assessment Tool (BAT) (ISTH-BAT), and their bleeding history was collected.

### 4.2. Blood Samples

Peripheral blood (PB) samples were collected by venipuncture into vacutainer tubes containing sodium citrate for PLT lumiaggregometry and FCM, and tripotassium ethylene diamine tetracetic acid (EDTA-K3) for blood counts, to perform genetic studies.

### 4.3. Blood Counts

Blood counts and PLT indexes were obtained using the automated hematological analyzer Sysmex XE-2100 (TOA Medical Electronics, Kobe, Japan).

### 4.4. Coagulation Tests and Platelet Functional Studies

Coagulation tests—prothrombin time (PT) and activated partial thromboplastin time (aPTT)—were performed in an ACLTOP (Werfen, Bedford, MA, USA). von Willebrand Factor Antigen (vWF:Ag), Ristocetin cofactor activity of von Willebrand factor (vWF:RCo), and factor VIII procoagulant activity (FVIII:C) dosing were performed in a BCSXP (Siemens, Marburg, Germany). The tests were performed according to each manufacturer’s instructions.

Occlusion tests were performed using the INNOVANCE PFA-200^®^ System (Siemens, Marburg, Germany), employing both collagen/epinephrine (COL/EPI) and collagen/adenosine diphosphate (COL/ADP) test cartridges and following the manufacturerߣs instructions.

Platelet aggregation and ATP release in response to different agonists—ADP, AA, COL, EPI, and TRAP-6, and PLT agglutination induced by RIST, were evaluated in a Chronolog 700 lumiaggregometer (Chrono-Log Corporation, Havertown, PA, USA) following manufacturerߣs instructions and using citrated platelet-rich plasma (PRP). All agonists were from Chrono-Log Corporation, Havertown, PA, USA except for TRAP-6 (Stago, Saint-Ouen-l’Aumône, France). ATP release was measured using the luciferin luciferase system (ChromoLume, Chrono-Log Corporation, Havertown, PA, USA). Light transmission and luminescence were recorded for at least 300 s. PRP from patients were analyzed in parallel with a control PRP from a pool of at least three healthy blood donors.

### 4.5. Flow Cytometry Studies

Flow cytometry studies were performed using a Navios™ flow cytometer from Beckman Coulter, Hialeah, FL, USA (BC).

Surface expression of PLT glycoproteins (GP) was measured by FCM using citrated PB and a direct immunofluorescence technique, as previously described in detail [12]. Monoclonal antibodies (mAbs) used, conjugated with fluorescein isothiocyanate (FITC) or with phycoerythrin (PE) were from Becton Dickinson, San Jose, CA, USA (BD) or from Dako, Glostrup, Denmark (DK): FITC-conjugated anti-CD41a (clone HIP8) (BD, 333147) FITC-conjugated anti-CD61 (GPIIIa) (clone RUU-PL7F12) (BD, 347407), and PE-conjugated anti-CD42b (GPIb) (clone AN51) (DK, R7014). Platelets were gated based on their forward scatter (FSC) and side scatter (SSC), and expression of the PLT-associated GP, and the mean fluorescence intensity (MFI) was measured for each platelet GP analyzed. The results were expressed as a percentage (%) of normal (patient MFI/median MFI of controls*100) and corrected for PLT size, as evaluated by the FSC.

Platelet ATP content was evaluated by FCM using mepacrine uptake. The results are presented as % of the normal values, calculated by dividing the median value of the MFI of the patient’s PLTs stained with mepacrine by the median of the MFI values obtained in three healthy controls studied in parallel.

Platelet activation was assessed in citrated whole blood by FCM, after stimulation with TRAP-6 (20 µM) or ADP (10 µM), as previously described in detail [12]. Monoclonal antibodies used were PE-conjugated anti-CD42b (GPIb) (clone AN51) (DK, catalog number R7014), FITC-conjugated PAC-1 (clone PAC-1) (BD, catalog number 340507) or FITC-conjugated antibFG (clone 9F9) (Biocytex, Marseille, France BX, catalog number 5009-F100T). Platelets were gated based on their FSC and SSC, and expression of CD42b, and analyzed for the expression of PAC-1 and bFG. The results are presented as % of the normal values, calculated by dividing the median value of the MFI of the patientߣs platelets stained with PAC-1 and anti-bFG after stimulation with TRAP-6 or ADP, by the mean of correspondent MFI values obtained in eight healthy controls studied in parallel.

### 4.6. Genetic Studies

After extraction of DNA from whole blood, genetic analysis of the index case was performed at the University of Cambridge (Cambridge, UK), using the ThromboGenomics high-throughput sequencing (HTS) platform, v2.8 (Table S1) [13]. Genetic analysis performed at our hospital uses a custom HTS panel including 92 genes related to hemostasis disorders that was designed using Ion Ampliseq designer software (Thermo Fisher Scientific, Waltham, MA, USA) (TFS) (Table S2). DNA library was prepared following Ion Ampliseq Library kit Plus instructions (TFS). Template preparation was performed in IonChef System, followed by sequencing in the Ion S5 system (TFS). After raw data analysis and variant calling in the Ion Torrent platform, variant annotation and filtering resorted to Ion Reporter v.5.14 software (TFS). A variant interpretation was performed using Alamut Visual v2.11 software (Interactive Biosoftware, Rouen, France), which includes algorithms to assess the impact of missense variants (SIFT (v6.2.0), MutationTaster2 (v2013) Polyphen-2(v2.2.2r39)) and effect on splicing [14,15,16].

Sanger sequencing was performed using primers designed to amplify exon 9 of *RASGRP2* and flanking intronic regions by PCR. After purification, sequencing PCR was performed with BigDye kit V3.1 (TFS). Sequencing electropherograms were compared with reference sequence NM_153819.1 resorting to SeqScape v2.5 software (TFS).

## Figures and Tables

**Table 1:** ISTH-BAT bleeding scores and laboratory results of the family cases.

Patients	V2	V1	V3	IV9	IV8	IV7
c.999G>C variant in *RASGRP2*	+/+	+/−	+/−	+/−	+/−	+/−
Bleeding score (ISTH-BAT)	8	5	2	6	6	7
Blood counts						
WBC (×10^9^/L)	7.05	4.21	6.99	7.54	5.3	9.6
Neutrophils (×10^9^/L)	2.76	2.4	3.33	4.50	2.68	5.89
Hb (g/dL)	13.9	16.3	13.7	14.7	12.5	16.7
Platelet (×10^9^/L)	268	195	277	277	293	230
MPV (fL)	10.6	8.9	11.3	9.5	11.7	12.1
Coagulation tests						
aPTT (s)	28.8	27.7	27.9	27.9	23.9	24.8
PT (s)	11.4	12.1	10.7	11.0	10.0	11.1
FVIII:C (%N)	109.8	134	137	120	NA	NA
vWF:Ag (%N)	72.8	127	151	77.6	NA	NA
vWF:RCo (%N)	65.7	125.4	124	51	NA	NA
PFA-200 (CT, s)						
COL/EPI	>288	123	78	207	88	69
COL/ADP	>278	77	115	131	79	78
Platelet aggregation (ATP release)						
COL (1 µg/mL), % (nmol)	59 (0.76)	77 (1.52)	71 (1.07)	88 (0.00)	83 (1.10)	95 (2.24)
ADP (10 µM), % (nmol)	0 (0.14)	75 (1.30)	87 (1.28)	0 (0.14)	59 (NA)	86 (1.64)
EPI (10 µM), % (nmol)	3 (0.00)	91 (1.46)	70 (1.17)	4 (0.00)	30 (0.00)	4 (0.00)
AA (1 mM), % (nmol)	2 (0.00)	78 (0.56)	74 (1.17)	84 (1.16)	79 (1.41)	74 (1.76)
TRAP-6 (25 µM), % (nmol)	75 (1.49)	81 (1.04)	84 (1.93)	85 (0.38)	86 (2.47)	95 (2.68)
Platelet agglutination						
RIST (1 mg/mL) (%)	33	91	72	22	79	85
Platelet glycoprotein levels						
GPIIb (CD41) (%N)	92	NA	NA	95	NA	NA
GPIIIa (CD61) (%N	125	NA	NA	99	NA	NA
GPIX (CD42a) (%N)	102	NA	NA	103	NA	NA
GPIbα (CD42b) (%N)	115	NA	NA	117	NA	NA
Platelet ATP content						
Mepacrine uptake (%N)	86	NA	NA	89	NA	NA
Platelet αIIbβ3 integrin activation						
TRAP-6/PAC-1 (%N)	28	92	NA	55	83	94
TRAP-6/bFG (%N)	23	138	NA	56	107	84
ADP/PAC-1 (%N)	30	99	NA	46	87	87
ADP/bFG (%N)	9	139	NA	34	64	64

## Data Availability

The authors confirm that the main data supporting the findings of this study are available within the article and its supplementary materials. More information is available from the corresponding author, upon reasonable request.

## Supplementary Materials

The following are available online at https://www.mdpi.com/article/10.3390/ijms222212423/s1.

## References

[1] Canault M., Ghalloussi D., Grosdidier C., Guinier M., Perret C., Chelghoum N., Germain M., Raslova H., Peiretti F., Morange P.E. (2014). Human CalDAG-GEFI gene (RASGRP2) mutation affects platelet function and causes severe bleeding. J. Exp. Med..

[2] Canault M., Alessi M.-C. (2020). RasGRP2 Structure, Function and Genetic Variants in Platelet Pathophysiology. Int. J. Mol. Sci..

[3] Chrzanowska-Wodnicka M., Smyth S.S., Schoenwaelder S.M., Fischer T.H., White G.C. (2005). Rap1b is required for normal platelet function and hemostasis in mice. J. Clin. Investig..

[4] Cifuni S.M., Wagner D.D., Bergmeier W. (2008). CalDAG-GEFI and protein kinase C represent alternative pathways leading to activation of integrin αIIbβ3 in platelets. Blood.

[5] Crittenden J.R., Bergmeier W., Zhang Y., Piffath C.L., Liang Y., Wagner D.D., E Housman D., Graybiel A.M. (2004). CalDAG-GEFI integrates signaling for platelet aggregation and thrombus formation. Nat. Med..

[6] Palma-Barqueros V., Ruiz-Pividal J., Bohdan N., Vicente V., Bastida J.M., Lozano M., Rivera J. (2019). RASGRP2 gene variations associated with platelet dysfunction and bleeding. Platelets.

[7] Westbury S., Canault M., Greene D., Bermejo E., Hanlon K., Lambert M.P., Millar C.M., Nurden P., Obaji S.G., Revel-Vilk S. (2017). Expanded repertoire of RASGRP2 variants responsible for platelet dysfunction and severe bleeding. Blood.

[8] Puetz J., Boudreaux M.K. (2011). Evaluation of the gene encoding calcium and diacylglycerol regulated guanine nucleotide exchange factor I (CalDAG-GEFI) in human patients with congenital qualitative platelet disorders. Platelets.

[9] Meller J., Malinin N., Panigrahi S., Kerr B., Patil A., Ma Y., Venkateswaran L., Rogozin I., Mohandas N., Ehlayel M.S. (2012). Novel aspects of Kindlin-3 function in humans based on a new case of leukocyte adhesion deficiency III. J. Thromb. Haemost..

[10] Manukjan G., Wiegering V.A., Reindl T., Strauß G., Klopocki E., Schulze H., Andres O. (2019). Novel variants in FERMT3 and RASGRP2-Genetic linkage in Glanzmann-like bleeding disorders. Pediatr. Blood Cancer.

[11] Bottega R., Pecci A., De Candia E., Pujol-Moix N., Heller P.G., Noris P., De Rocco D., Podda G.M., Glembotsky A.C., Cattaneo M. (2012). Correlation between platelet phenotype and NBEAL2 genotype in patients with congenital thrombocytopenia and α-granule deficiency. Haematologica.

[12] Morais S., Oliveira J., Lau C., Pereira M., Gonçalves M., Monteiro C., Gonçalves A.R., Matos R., Sampaio M., Cruz E. (2020). αIIbβ3 variants in ten families with autosomal dominant macrothrombocytopenia: Expanding the mutational and clinical spectrum. PLoS ONE.

[13] Downes K., Megy K., Duarte D., Vries M., Gebhart J., Hofer S., Shamardina O., Deevi S.V.V., Stephens J., Mapeta R. (2019). Diagnostic high-throughput sequencing of 2396 patients with bleeding, thrombotic, and platelet disorders. Blood.

[14] Sim N.-L., Kumar P., Hu J., Henikoff S., Schneider G., Ng P.C. (2012). SIFT web server: Predicting effects of amino acid substitutions on proteins. Nucleic Acids Res..

[15] Schwarz J.M., Cooper D.N., Schuelke M., Seelow D. (2014). MutationTaster2: Mutation prediction for the deep-sequencing age. Nat. Methods.

[16] Adzhubei I.A., Schmidt S., Peshkin L., Ramensky V.E., Gerasimova A., Bork P., Kondrashov A.S., Sunyaev S.R. (2010). A method and server for predicting damaging missense mutations. Nat. Methods.

