# Supplementary material for: CalDAG-GEFI Deficiency in a Family with Symptomatic Heterozygous and Homozygous Carriers of a Likely Pathogenic Variant in RASGRP2
Source: Int J Mol Sci. 2021 Nov 17;22(22):12423. doi: 10.3390/ijms222212423 (PMC8618213; doi:10.3390/ijms222212423)
Supplement: Supplementary file 1 [file ijms-22-12423-s001.zip › ijms-1426381-supplementary.pdf]

International Journal of *Molecular Sciences*

MDPI

Article

CalDAG-GEFI deficiency in a family with symptomatic heterozygous and homozygous carriers of a likely pathogenic variant in *RASGRP2*.

Sara Morais[1,2], Mónica Pereira[1,2], Catarina Lau[2,3], Ana Gonçalves[2,4], Catarina Monteiro[1,2], Marta Gonçalves[2,3], Jorge Oliveira[4], Lurdes Moreira[1], Eugénia Cruz[1], Rosário Santos[2,4] and Margarida Lima[2,3]

Table S1. Genes included in the ThromboGenomics HTS platform v2.8

ACTN1	*ANKRD26*	*ANO6*	*AP3B1*	*BLOC1S3*	*BLOC1S6*	*CHST14*	*CYCS*
DIAPH1	*DTNBP1*	*ETV6*	*F2*	*F5*	*F7*	*F8*	*F9*
F10	*F11*	*F13A1*	*F13B*	*FERMT3*	*FGA*	*FGB*	*FGG*
FLI1	*FLNA*	*GATA1*	*GFI1B*	*GGCX*	*GNE*	*GP1BA*	*GP1BB*
GP6	*GP9*	*HOXA11*	*HPS1*	*HPS3*	*HPS4*	*HPS5*	*HPS6*
HRG	*ITGA2B*	*ITGB3*	*LMAN1*	*LYST*	*MCFD2*	*MPL*	*MYH9*
NBEA	*NBEAL2*	*ORAI1*	*P2RY12*	*PLA2G4A*	*PLG*	*PLAT*	*PLAU*
PROC	*PROS1*	*RASGRP2*	*RBM8A*	*RUNX1*	*SERPINC1*	*SERPIND1*	*SERPINE1*
SERPINF2	*STIM1*	*STXBP2*	*TBXAS1*	*TBXA2R*	*THBD*	*THPO*	*VIPAS39*
VKORC1	*VPS33B*	*VWF*	*WAS*				

Table S2. Genes included in our HTS platform for molecular screening of hemostasis disorders designed using Ion Ampliseq software (Thermo Fisher Scientific).

ABCA1	*ABCG5*	*ABCG8*	*ACTN1*	*ADAMTS13*	*ANKRD26*	*ANO6*	*AP3B1*
AP3D1	*ARPC1B*	*BLOC1S6*	*CD36*	*COL1A1*	*COL5A1*	*COL5A2*	*CYCS*
DHCR24	*DIAPH1*	*DPAGT1*	*ETV6*	*F13A1*	*F13B*	*FGA*	*FGB*
FGG	*FLNA*	*GATA1*	*GFI1B*	*GGCX*	*GNAI3*	*GNAQ*	*GNAS*
GNE	*GP1BA*	*GP5*	*GP6*	*HOXA11*	*HPS1*	*HPS3*	*HPS4*
HPS5	*HPS6*	*HRG*	*ITGA2*	*ITGA2B*	*ITGB3*	*KNG1*	*LMAN1*
LYST	*MASTL*	*MCFD2*	*MECOM*	*MPL*	*MYH9*	*MYO5A*	*NBEA*
NBEAL2	*P2RX1*	*P2RY1*	*P2RY12*	*PLA2G4A*	*PLAT*	*PLAU*	*PRF1*
PTGS1	*PTPN11*	*PTS*	*RAB27A*	*RASGRP2*	*RBM8A*	*RGS2*	*RUNX1*
SERPINC1	*SERPIND1*	*SERPINE1*	*SERPINF2*	*SLC45A2*	*STIM1*	*STX11*	*STXBP2*
TBXAS1	*THBD*	*THPO*	*TUBB1*	*UNC13D*	*USF1*	*USF1*	*VIPAS39*
VKORC1	*VPS33B*	*VWF*	*WAS*				
